# Integrin-β1, not integrin-β5, mediates osteoblastic differentiation and ECM formation promoted by mechanical tensile strain

**DOI:** 10.1186/s40659-015-0014-y

**Published:** 2015-05-14

**Authors:** Qiangcheng Zeng, Yong Guo, Yongming Liu, Ruixin Li, Xinchang Zhang, Lu Liu, Yang Wang, Xizheng Zhang, Xianqiong Zou

**Affiliations:** Shandong Provincial Key Laboratory of Functional Macromolecular Biophysics, Institute of Biophysics, Dezhou University, Dezhou, 253023 Shandong China; College of Biotechnology, Guilin Medical University, Guilin, 541004 Guangxi China; Institute of Medical Equipment, Academy of Military Medical Sciences, Tianjin, 300161 China; Chemistry Department, Logistics College of Chinese People’s Armed Police Forces, Tianjin, China

**Keywords:** Tensile strain, Osteoblast, Extracellular matrix, Integrin, siRNA

## Abstract

**Background:**

Mechanical strain plays a great role in growth and differentiation of osteoblast. A previous study indicated that integrin-β (β1, β5) mediated osteoblast proliferation promoted by mechanical tensile strain. However, the involvement of integrin-β in osteoblastic differentiation and extracellular matrix (ECM) formation induced by mechanical tensile strain, remains unclear.

**Results:**

After transfection with integrin-β1 siRNA or integrin-β5 siRNA, mouse MC3T3-E1 preosteoblasts were cultured in cell culture dishes and stimulated with mechanical tensile strain of 2500 microstrain (με) at 0.5 Hz applied once a day for 1 h over 3 or 5 consecutive days. The cyclic tensile strain promoted osteoblastic differentiation of MC3T3-E1 cells. Transfection with integrin-β1 siRNA attenuated the osteoblastic diffenentiation induced by the tensile strain. By contrast, transfection with integrin-β5 siRNA had little effect on the osteoblastic differentiation induced by the strain. At the same time, the result of ECM formation promoted by the strain, was similar to the osteoblastic differentiation.

**Conclusion:**

Integrin-β1 mediates osteoblast differentiation and osteoblastic ECM formation promoted by cyclic tensile strain, and integrin-β5 is not involved in the osteoblasts response to the tensile strain.

**Electronic supplementary material:**

The online version of this article (doi:10.1186/s40659-015-0014-y) contains supplementary material, which is available to authorized users.

## Background

Mechanical forces are important regulators of bone homeostasis [1]. Application of mechanical loading promotes bone formation, whereas lack of loading results in bone loss [2]. Osteoblasts and their precursor cells are sensitive to mechanical strain. Many studies have shown that mechanical strains are crucial for the regulation of osteoblastic proliferation, differentiation, and apoptosis [3,4].

Integrins act as mechanotransducers and the stimulation of them has been shown to regulate cellular growth and gene expression [5]. In bone, integrins transduce mechanical signals imposed on bone into responses of bone cells [6,7]. Integrin-β1 plays great roles in osteoblasts^,^ activity, expression of an osteoblast-specific dominant negative form of integrin-β1 resulted in reduced bone mass [8]. Fluid flow shear stress increased integrin-β1 expression of osteoblasts [9], and the shear stress induced osteogenic differentiation through integrin-β1 [10].

Mechanical tensile strain induced the expressions of integrin-β1 and integrin-β5 of MC3T3-E1 preosteoblasts [11], and increased the expression of ECM-related proteins, such as osteonectin, osteopontin (OPN), osteocalcin (OCN), and collagen type I (Col I) [12]. In addition, mechanical tensile strain (0.8-3.2%) induced human osteoblastic differentiation [13].

Previous studies indicated that mechanical tensile strain promoted osteoblastic differentiation and ECM production of MC3T3-E1 preosteoblasts [14,15], integrin-β1 and integrin-β5 were involved in osteoblasts proliferation regulated by the cyclic tensile strain [11]. However, the involvement of integrin- β1 and β5 in osteoblastic differentiation and ECM formation induced by mechanical tensile strain, remains unclear. In this study, MC3T3-E1 cells were stimulated with the mechanical tensile strain, then the mediation of integrin-β1 and β5 in osteoblastic differentiation and ECM formation promoted by the tensile strain, was investigated.

## Results

### Cyclic tensile strain promotes osteoblastic differentiation and ECM formation

**Figure 1:**
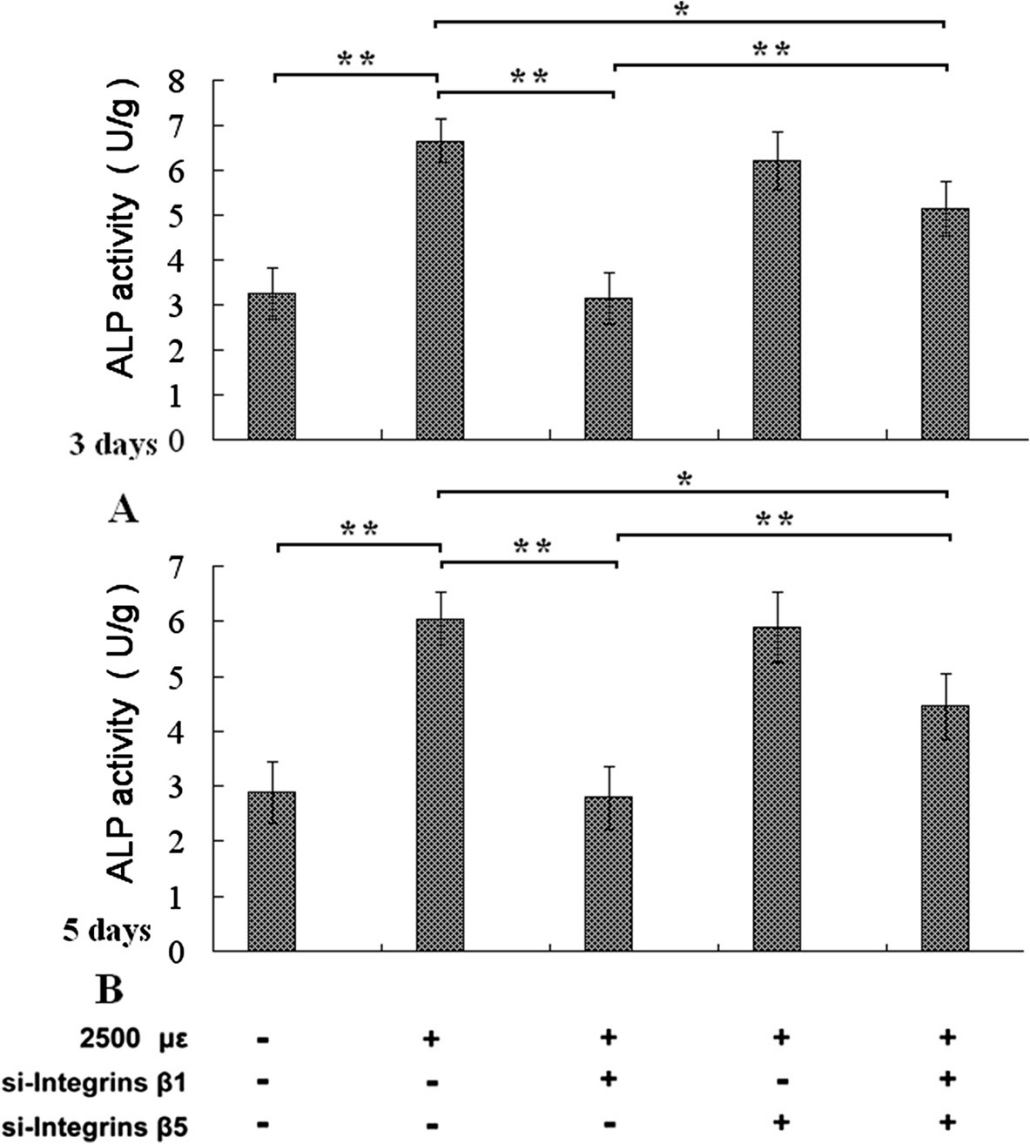
Assay of ALP activity. The results showed that the mechanical tensile strain of 2500 με at 0.5 Hz applied 1 h per day for 3 days **(A)** or 5 days **(B)**, elevated ALP activity of pre-osteoblastic MC3T3-E1 cells, pretreatment of integrin-β1 siRNA inhibited the mechanical strain induced elevation of ALP activity (the first and second column of **A** and **B**). Integrin-β5 siRNA had little effect on ALP activity, and pretreatments of both integrin-β1 siRNA and integrin-β5 siRNA simultaneously also attenuated the elevation of ALP activity, but the effect was weaker than only integrin-β1 siRNA. *P < 0.05, **P < 0.01, between indicated groups.

**Figure 2:**
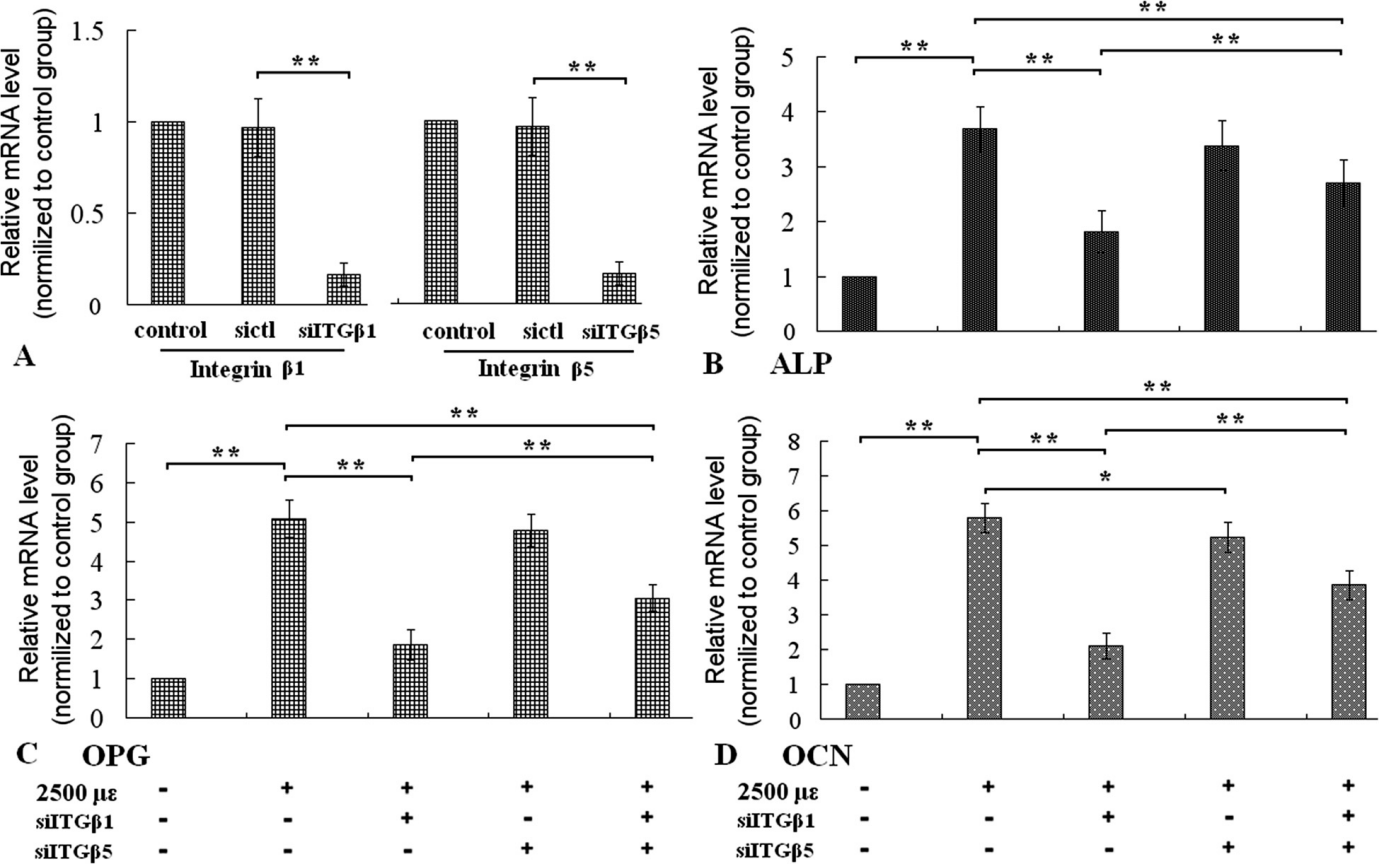
Assay of mRNA levels of integrin-β1, integrin-β5, ALP, OCN, and OPG using real-time PCR. Pretreatment of integrin-β1 or ntegrin-β5 siRNA reduced mRNA level of integrin-β1 or β5 **(A)**. The mechanical tensile strain of 2500 με enhanced mRNA levels of ALP, OCN, and OPG in MC3T3-E1 cells (the first and second column of **B**, **C** and **D**), pretreatment of integrin-β1 siRNA inhibited the mechanical induced the enhancement of the three mRNAs levels. Integrin-β5 siRNA had little effect on these mRNA levels, and pretreatments of both integrin-β1 siRNA and integrin-β5 siRNA simultaneously also attenuated the elevation, but the inhibitory effect was weaker than only integrin-β1 siRNA. *P < 0.05, **P < 0.01, between indicated groups.

**Figure 3:**
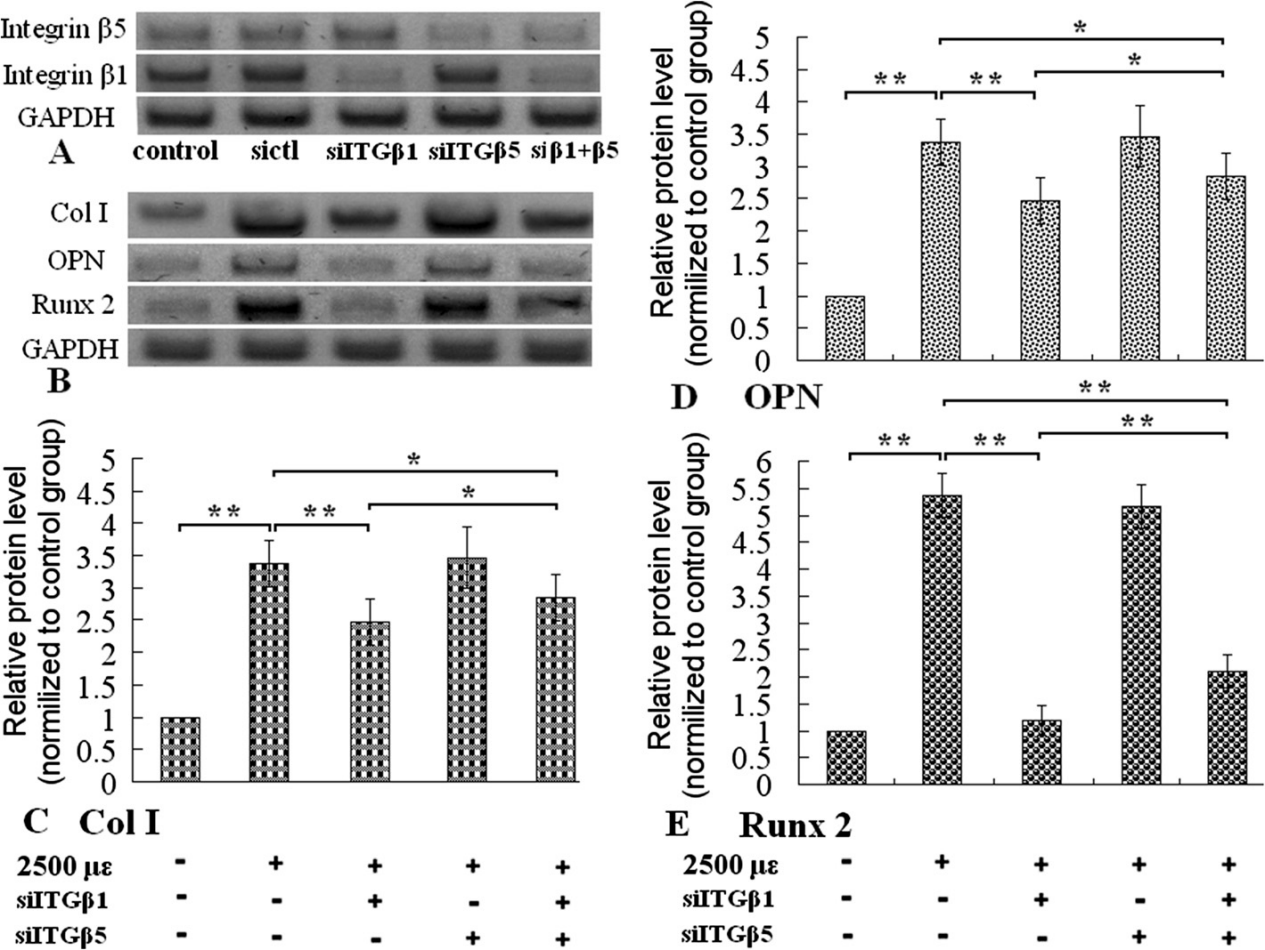
Western Blot analysis of protein levels of integrin-β1 and β5, Runx 2, OPN and Col I. Integrin-β1 or integrin-β5 siRNA reduced protein level of integrin-β1 or integrin-β5 **(A)**. B was the image of Western Blot analysis of Col I, OPN, Runx 2. The mechanical tensile strain increased protein levels of Runx 2, OPN and Col I (the first and second column of **C**, **D** and **E**), pretreatment of integrin-β1 siRNA inhibited the tensile strain induced the increment of the three proteins levels. Knockdown of integrin-β5 had little effect on these protein levels, and knockdown of both integrin-β1 and integrin-β5 simultaneously also attenuated the elevation, but the inhibitory effect was less than only integrin-β1 knockdown. *P < 0.05, **P < 0.01, between indicated groups.

**Figure 4:**
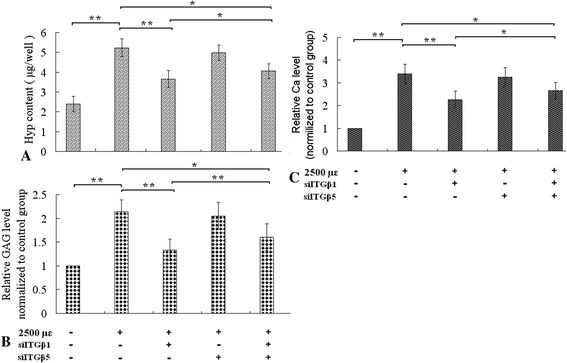
Assay of Hydroxyproline (Hyp), GAG and Calcium (Ca) in ECMs. The mechanical strain increased Hyp content, and relative levels of GAG and Ca (the first and second column of **A**, **B** and **C**). Integrin-β1 siRNA reduced the content of Hyp, levels of GAG and Ca, which increased by the tensile strain. The effect of integrin-β5 siRNA on levels of hyp, GAG and Ca was nearly naught, the inhibitory effect of both integrin-β1 siRNA and integrin-β5 siRNA simultaneously on hyp, GAG, Ca was weaker than only integrin-β1 siRNA. *P < 0.05, **P < 0.01, between indicated groups.

After exposing the MC3T3-E1 cells to mechanical tensile strain of 2500 με at 0.5 Hz, 1 h/day, for 3 days (or 5 days), the ALP activity was enhanced (Figure 1, the first, and the second column of A and B panels), the mRNA levels of *ALP*, *OCN*, and *OPG* were all elevated (Figure 2, the first, and the second column of B, C, D panels). The cyclic tensile strain for 3 days increased protein levels of Runx 2, OPN, Col I (Figure 3, the first, and the second column of A-E panels), and Ca content of ECM (Figure 4, the first, and the second column of C panels). ALP and OPN are markers of early osteoblastic differentiation [16,17]. OCN, OPG, Runx 2, Col I, and Ca content are all markers for osteoblastic differentiation in previous study [14,15,18,19]. Therefore, these results indicated that the mechanical tensile strain promoted osteoblastic differentiation.

Additionally, the tensile strain increased the hydroxyproline, GAG content of the ECM which was coated on loading dishes (Figure 4, the first, and the second column of A and B panels). Resulting hydroxyproline measurements were converted to collagen contents following a 1: 10 (hydroxyproline: collagen) ratio [20], the levels of hydroxyproline equated to the collagen content. GAG and collagen are main components of ECM, and Col I is a kind of main collagen, so that the tensile strain increased ECM production of osteoblasts.

### nvolvement of integrin-β in the accelerating effects of cyclic tensile strain on osteoblastic differentiation and ECM formation

After knockdown of integrin-β1 or β5 with siRNA, we assayed ALP activity, the expression levels of ALP, OCN, OPG, Runx 2, OPN, Col I of MC3T3-E1 cells, and Ca content of ECM. Transfection with integrin-β1 siRNA inhibited the enhancement of ALP activity stimulated by the mechanical tensile strain (Figure 1), integrin-β1 siRNA also attenuated increment of mRNA levels of ALP, OCN, OPG, protein levels of Runx 2, OPN, Col I (Figures 2 and 3), and Calcium content (Figure 4). Knockdown of integrin-β5 had little effect on osteoblastic differentiation (Figures 1, 2 and 3). The results indicated that integrin-β1, not integrin-β5, was involved in the accelerating effect of the mechanical tensile strain on osteoblastic differentiation.

After knockdown of integrin-β1 or β5, we assayed hydroxyproline and GAG of ECM. Transfection with integrin-β1 siRNA lowered contents of hydroxyproline and GAG, which increased by mechanical tensile strain, and knockdown of integrin-β5 hardly affected increment of hydroxyproline and GAG (Figure 4). The results showed that integrin-β1, not integrin-β5, was involved in the accelerating effect of the tensile strain on ECM formation.

Notablely, simultaneous knockdowns of both integrin-β1 and β5 inhibited the two kinds of accelerating effects, but the inhibitory effect was weaker than knockdowns of integrin-β1 only (Figures 1, 2, 3 and 4).

In unstrained cells, knocking down integrin-β1, integrin-β5 or both of them, nearly had no effect on protein expressions of OPN and Runx 2, and hydroxyproline level of ECM. Integrin-β1 siRNA reduced OCN mRNA expression and ALP activity, but the effect was weak. Integrin-β5 siRNA or knocking down both of integrin-β1 and β5 had no effect on OCN mRNA expression and ALP activity (Additional file 1: Figure S2). The results showed: the osteoblastic differentiation and ECM formation did not change significantly without mechanical tensile strain, even the cells were pretreated with integrin-β1 or integrin-β5 siRNA.

## Discussion

Bone optimizes its load-bearing role by adapting its architecture and function to mechanical loading. Lack of mechanical strain causes loss of bone mass while a suitable dynamic mechanical strain promotes bone formation [21,22]. Mechanical strain can induce bone remodeling activity resulting in structural changes. In bone, mechanical strain are transmitted through ECM to resident osteoblasts, osteocytes, periosteal cells and osteoclasts [23].

Osteoblasts are important mechanical receptors that can transform mechanical stimuli into biochemical signals and secrete bone matrix to promote bone matrix mineralization [24]. Previous investigations showed that mechanical strain increased matrix mineralization of osteoblasts [24,25] enhanced expressions of bone ECM-related proteins/genes [12,16], and induced osteoblasts differentiation [13]. Previous studies also indicated that mechanical tensile strain (at a frequency of 0.5 Hz and intensities of 2000–3000 με for 1 h/day) promoted osteoblastic differentiation [15,23,26], and promoted formation of the osteoblast ECM [14]. Consequently, in this study, we selected the mechanical tensile strain (0.5 Hz, 1 h/day) to stimulate MC3T3-E1 cells. Our study indicated that the cyclic tensile strain promoted osteoblast differentiation and increased osteoblastic ECM, which confirmed that our selection was appropriate.

Integrins are major family of cell-surface receptors, they are transmembrane heterodimers comprised of α- and β-subunits [27]. The extracellular domain of integrin interacts with ECM proteins, including collagen, fibronectin, laminin, and vitronectin. The intracytoplasmic domain interacts with intracellular signal transmission molecules and cytoskeletal proteins to regulate signal transduction, cytoskeletal remodeling, cell motility, migration, apoptosis, cell proliferation and cell adhesion [28,29]. Integrins provide a preferred site for mechanical signal transfer across the cell surface and transmit the signal from the plasma membrane to the cytoskeleton [30].

Mechanical tensile strain up-regulated the expression of integrin-β1 in osteosarcoma cells [31], and fluid shear stress (20 dynes/cm^2^, 30 min) increased the expression of integrin-β1 in C57BL/6 J mouse osteoblasts [32]. In addition, the mechanical tensile strain up-regulated the expressions of integrin-β1 and β5, the two kinds of integrin subunits were involved in osteoblast proliferation regulated by mechanical strain [11]. Therefore, these previous studies induced us to investigate the involvement of integrin-β1 and β5 in osteoblastic differentiation and ECM formation promoted by mechanical tensile strain.

In this study, knockdown of integrin-β1 inhibited osteoblastic differentiation and ECM formation which were promoted by mechanical strain, and knockdown of integrin-β5 had little effect on osteoblastic differentiation and ECM formation. The results demonstrate that integrin-β1, not integrin-β5, is involved in mechanical strain promoted osteoblastic differentiation and ECM formation.

More interestingly, in this study, simultaneous knockdowns of both integrin-β1 and β5 also inhibited osteoblastic differentiation and ECM formation promoted by mechanical strain, but the inhibitory effect was weaker than only integrin-β1 knockdown. The results indicate that knockdown of integrin-β5 attenuate the inhibitory effect of integrin-β1 knockdown on osteoblastic differentiation and ECM formation. Therefore, it would be more interesting to investigate the relationship between integrin-β1 and β5 in the future.

## Conclusion

In summary, we conclude that integrin-β1 mediates osteoblast differentiation and osteoblastic ECM formation promoted by mechanical tensile strain, and integrin-β5 is not involved in the mechanical response of osteoblasts. However, knockdown of integrin-β5 attenuated the inhibitory effect of integrin-β1 knockdown on osteoblastic differentiation and ECM formation.

## Methods

### Cell culture and application of mechanical strain

MC3T3-E1 cells, a mouse pre-osteoblastic cell line,were transferred to mechanical loading dishes that were reformed from cell culture dishes (Nunc International, Roskilde, Denmark), then maintained in alpha minimal essential medium (α-MEM; Invitrogen, San Diego, CA, USA) supplemented with 10% fetal calf serum and 1% penicillin-streptomycin (Hyclone, Logan, UT, USA).

At confluence, the MC3T3-E1 cells were subjected to mechanical tensile strain of 2500 με at 0.5 Hz for 1 h/day at indicated times. The cyclic tensile strain was generated by a specially designed four-point bending device, as previously described [33]. The device was driven by a stepping motor (controlled by a single chip microcomputer), and has been shown to produce homogenous, uniaxial strains to the substrate of mechanical loading dishes so that in the loading dishes, every cell is subjected to the same mechanical tensile strain [34,35].

### Alkaline phosphatase (ALP) activity assay

After mechanical strain, cells were lysed by brief sonication in the lysis buffer (10 mmol/L, HEPES, 250 mmol/L sucrose, 5 mmol/L Tris–HCl, 0.1% TritonX-100, pH 7.5). The ALP activity of the lysates was measured with ALP Activity Assay Kit (Nanjing Jiancheng Biotechnology Co. Ltd, China) at 25°C using the p-nitrophenyl phosphate method according to manufacturer’s protocol. ALP activity of each sample was normalized to protein concentration.

### Real-time polymerase chain (PCR) reaction

Total RNA was extracted with Trizol reagent (Invitrogen), then the cDNA was synthesised using the Rever TraPlus Kit (Toyobo Co., Ltd., Osaka, Japan). Real-time PCR was performed to detect mRNA levels of *ALP*, *OCN*, *OPG* and *GAPDH* (internal control reference) using SYBR Green I PCR Mix (Real SYBR Mixture, Beijing Cowin Biotech Co., Ltd. Beijing, China) on an Real-Time PCR System (7900; Applied Biosystems, CA, USA) according to the manufacturer’s instructions. Primer sequences are listed in Table 1. The amplification reaction included a denaturation step at 94°C for 180 s followed by 40 cycles of 94°C for 15 s, and at each annealing temperature for 30 s. Using the relative quantitative method (2^-ΔΔCt^), the levels of the PCR products of interest were calculated relative to those control group.

**Table 1:** **Sequences of primers used for Real-time PCR**

**Gene**	**Primer sequence (5′-3′)**	**Length (bps)**
ITGβ1	**F:** GCAACGCATATCTGGAAACA; **R**: CAAAGTGAAACCCAGCTACC	140
ITGβ5	**F**:TCCTGCTTCGAGAGTGAGT; **R**: CCTGCGTGGCATTTGCATT	137
ALP	**F**: CGGGACTGGTACTCGGATAA; **R**: ATTCCACGTCGGTTCTGTTC	157
OCN	**F**:AGTCTGACAAAGCCTTCA; **R**:AAGCAGGGTTAAGCTCACA	134
OPG	**F**:AGTCTGAGGAAGACCATGAG; **R**:AAACAGCCCAGTGACCATTC	205
GAPDH	**F:** ACCCATCACCATCTTCCAGGAG; **R**: GAAGGGGCGGAGATGATGAC	159

### Western blot analysis

The cell lysates were prepared in RIPA lysis buffer (Beyotime Institute of Biotechnology, Nantong, China). Protein concentration was quantified using Brandford’s method. Equal amounts of proteins were separated by electrophoresis on a polyacrylamide gel containing 0.1% SDS, then electrotransferred onto PVDF membranes (Millipore, Bedford, MA, USA). After blocking with 5% skim milk and incubation with primary antibodies respectively, the membranes were incubated with horseradish peroxidase-conjugated secondary antibody. The immunoreactive bands were visualized using an ECL detection kit (Wuhan Boster Bioengineering Co. Ltd, China). Glyceraldehyde3-phosphatedehydrogenase (GAPDH) was used as a loading control, data were normalized against those of corresponding optical density of GAPDH.

### Assay of ECM

After 5 days of mechanical stimulus, the MC3T3-E1 cells were eliminated according to our established method [36,37] (showed in Additional file 1), then the ECMs coated on the loading dishes were prepared (Additional file 1: Figure S1).

A. Measure hydroxyproline content. The ECMs on the loading dishes were hydrolyzed with hydrolysis buffer and the hydroxyproline content were detected with the Chloramine-T Hydroxyproline Assay Kit contain lysis buffer (Nanjing Jiancheng Biotechnology Institute Co., Ltd., Nanjing, China.) according to the manufacturer’s protocol.
B. Glycosaminoglycan (GAG) analysis. The ECMs were lysed with lysis buffer and GAG levels were assayed with Dimethyl-methylene Blue GAG Assay Kit contain lysis buffer (Xianmen Maiwei Biotechnology Co., Ltd., Xiamen, China.). The absorbance or optical density (OD) at 660 nm was regarded as relative level of GAG in the ECMs. Results were expressed as relative to control group (the ECM of unstrained cells on loading dishes).
C. Calcium deposition assay. After the ECMs were treated overnight with 0.1 M HCl, the ECM-deposited calcium content of the dishes was measured with the Calcium Assay Kit (Nanjing Jiancheng Biotechnology Co., Inc.) using the methylthymol blue complexon method according to the manufacturer’s instructions.

### RNA interfering (RNAi) against integrin-β1 and integrin-β5

Small interfering RNA (siRNA) targeting mouse integrin-β1 (siITGβ1) and integrin-β5 (siITGβ5) was obtained from Invitrogen. MC3T3-E1 cells (70%-80% confluence) were transfected with siITGβ1 (siITGβ5) or negative control siRNA (siCtl) using High-Fect Transfection Reagent (Beijing Cowin Bioscience Co., Ltd., Beijing, China), according to the manufacturer’s recommendations. Two days after transfection and 3 days (or 5 days) of mechanical stimulus, following experiments (ALP activity assay, real-time PCR, Western blot, and ECM assays) were performed. Table 2 lists the small RNA sequences. In order to investigate the relationship between integrin-β1 and β5, simultaneous knockdown of both integrin-β1 and β5 with siRNA was performed.

**Table 2:** **Small interfering RNA sequences**

**Description**	**Type**	**Sequence (5′-3′)**
ITGβ1	RNA	UAGAAAUGUUGGAACACUUUCGUCC
		GGACGAAAGUGUUCCAACAUUUCUA
ITGβ5	RNA	UACAGCCGCAUGUGCAAUUGUAGGC
		GCCUACAAUUGCACAUGCGGCUGUA

### Statistical analysis

All experiments were performed in triplicate and repeated at least three times. All data are showed as mean ± SD and were analyzed with one-way ANOVA followed by SNK pairwise comparisons. Statistical analysis was performed using SPSS software version 13.0. The p values <0.05 were considered statistically significant.

## Additional file

Additional file 1: Figure S1. Preparation of osteoblast ECM which was coated on dishes. A. Osteoblasts were observed via inverted microscopy. The cells were removed after treatment with PBS containing 0.5% Triton X-100 and NH_4_OH, and treated with 100 units/ml DNase, then the ECMs formed on the surfaces were revealed. B. The cells were stained with DAPI Staining Solution (Wuhan Boster Bioengineering Co., Ltd, Wuhan, China), according to manufacturer’s protocol. Before decellularization, the nucleus were observed with fluorescence microscope. After decellularization, the nucleus were removed. **Figure S2.** The protein expression of OPN and Runx 2, the ALP activity and mRNA expression of OCN in unstrained cells, and the relative hydroxyproline (Hyp) level of unstrained cells. Pretreatment of integrin-β1 siRNA or integrin-β5 siRNA nearly had no effect on protein expressions of OPN and Runx 2, and the hydroxyproline level. Integrin-β1 siRNA reduced OCN mRNA expression and ALP activity, but the effect was weak.

## Abbreviations

ECM: Extracellular matrix
με: Microstrain
ALP: Alkaline phosphatase
OCN: Osteocalcin
OPG: Osteoprotegerin
Runx 2: Runt-related transcriptional factor 2
OPN: Osteopontin
Col I: Collagen type I
GAG: Glycosaminoglycan
α-MEM: Alpha minimal essential medium
PCR: Polymerase chain reaction
GAPDH: Glyceraldehyde3-phosphatedehydrogenase
siRNA: Small interfering RNA

## References

[1] Ruimerman RP, Hilbers P, van Rietbergen B, Huiskes R (2005). A theoretical framework for strain-related trabecular bone maintenance and adaptation. J Biomech.

[2] Martin RB (2000). Toward a unifying theory of bone remodeling. Bone.

[3] Kaneuji T, Nogami S, Ariyoshi W, Nishihara T, Takahashi T (2011). Regulatory effect on osteoclastogenesis of mechanical strain-loaded osteoblasts. Int J Oral Maxillofac Surg.

[4] Rumney RM, Sunters A, Reilly GC, Gartland A (2012). Application of multiple forms of mechanical loading to human osteoblasts reveals increased ATP release in response to fluid flow in 3D cultures and differential regulation of immediate early genes. J Biomech.

[5] Brancaccio M, Fratta L, Notte A, Hirsch E, Poulet R, Guazzone S (2003). Melusin, a muscle specific integrin beta1-interacting protein, is required to prevent cardiac failure in response to chronic pressure overload. Nat Med.

[6] Pommerenke H, Schmidt C, Dürr F, Nebe B, Lüthen F, Muller P (2002). The mode of mechanical integrin stressing controls intracellular signaling in osteoblasts. J Bone Miner Res.

[7] Shyy JY, Chien S (2002). Role of integrins in endothelial mechanosensing of shear stress. Circ Res.

[8] Zimmerman D, Jin F, Leboy P, Hardy S, Damsky C (2000). Impaired bone formation in transgenic mice resulting from altered integrin function in osteoblasts. Dev Biol.

[9] Kapur S, Baylink DJ, Lau KH (2003). Fluid flow shear stress stimulates human osteoblast proliferation and differentiation through multiple interacting and competing signal transduction pathways. Bone.

[10] Mai Z, Peng Z, Wu S, Zhang J, Chen L, Liang H (2013). Single bout short duration fluid shear stress induces osteogenic differentiation of mc3t3-e1 cells via integrin b1 and BMP2 signaling cross-talk. PLoS One.

[11] Yan YX, Gong YW, Guo Y, Lv Q, Guo C, Zhuang Y (2012). Mechanical strain regulates osteoblast proliferation through integrin mediated ERK activation. PLoS One.

[12] Bhatt KA, Chang EI, Warren SM, Lin SE, Bastidas N, Ghali S (2007). Uniaxial mechanical strain: an in vitro correlate to distraction osteogenesis. J Surg Res.

[13] Zhu J, Zhang X, Wang C, Peng X, Zhang X (2008). Different magnitudes of tensile strain induce human osteoblasts differentiation associated with the activation of ERK1/2 phosphorylation. Int J Mol Sci.

[14] Guo Y, Zhang CQ, Zeng QC, Li RX, Liu L, Hao QX (2012). Mechanical strain promotes osteoblast ECM formation and improves its osteoinductive potential. Biomed Eng Online.

[15] Liu L, Guo Y, Wan Z, Shi C, Li J, Li R (2012). Effects of phytoestrogen a-ZAL and mechanical stimulation proliferation, osteoblastic differentiation, and OPG/RANKL expression in MC3T3-E1 pre-osteoblasts. Cell Mol Bioeng.

[16] Di Palma F, Douet M, Boachon C, Guignandon A, Peyroche S, Forest B (2003). Physiological strains induce differentiation in human osteoblasts cultured on orthopaedic biomaterial. Biomaterials.

[17] Beck GR, Zerler B, Moran E (2000). Phosphate is a specific signal for induction of osteopontin gene expression. Proc Natl Acad Sci U S A.

[18] Li J, Wan Z, Liu H, Li H, Liu L, Li R (2013). Osteoblasts subjected to mechanical strain inhibit osteoclastic differentiation and bone resorption in a co-culture system. Ann Biomed Eng.

[19] Guo Y, Zeng Q, Yan Y, Shen L, Liu L, Li R (2013). Proliferative effect and osteoinductive potential of extracellular matrix coated on cell culture plates. Springerplus.

[20] Patra C, Talukdar S, Novoyatleva T, Velagala SR, Mühlfeld C, Kundu B (2012). Silk protein fibroin from antheraea mylitta for cardiac tissue engineering. Biomaterials.

[21] Hillam RA, Skerry TM (1995). Inhibition of bone resorption and stimulation of formulation by mechanical loading of the modeling rat ulna in vivo. J Bone Miner Res.

[22] Rubin CT, Lanyon LE (1984). Regulation of bone formation by applied dynamic loads. J Bone Joint Surg Am.

[23] Yan YX, Song M, Guo C, Guo Y, Gong YW, Li RX (2010). The effects of substrate-streching strain on theBMP-2 mRNA expression in three kinds of mouse cell lines. Chin J Gerontol.

[24] Wozniak M, Fausto A, Carron CP, Meyer DM, Hruska KA (2000). Mechanically strained cells of the osteoblast lineage organize their extracellular matrix through unique sites of alphavbeta3-integrin expression. J Bone Miner Res.

[25] Simmons CA, Matlis S, Thornton AJ, Chen S, Wang CY, Mooney DJ (2003). Cyclic strain enhances matrix mineralization by adult human mesenchymal stem cells via the extracellular signal-regulated kinase (ERK1/2) signaling pathway. J Biomech.

[26] Gong YW, Yan YX, Zhang Y, Zhang XZ, Guo Y (2011). The effect of substrate-stretching strain on the expression of Runx2 in mouse osteoblasts. Chin J Osteoporos.

[27] Ingber DE (2003). Tensegrity I, cell structure and hierarchical systems biology. J Cell Sci.

[28] Edwin AC, Joan SB (1995). Integrins and signal transduction pathways: the road taken. Science.

[29] Giancotti FG, Ruoslahti E (1999). Integrin signaling. Science.

[30] Yutao X, Geru W, Xiaojun B, Tao G, Aiqun M (2006). Mechanical stretch induced hypertrophy of neonatal rat ventricular myocytes is mediated by β1-integrin- microtubule signaling pathways. Eur J Heart Fail.

[31] Carvalho RS, Scott JE, Yen EH (1995). The effects of mechanical stimulation on the distribution of beta 1-integrin and expression of beta 1-integrin mRNA inTE-85 human osteosarcoma cells. Arch Oral Biol.

[32] Lau KH, Kapur S, Kesavan C, Baylink DJ (2006). Up-regulation of the Wnt, estrogen receptor, insulin-like growth factor-I, and bone morphogenetic protein pathways in C57BL/6 J osteoblasts as opposed to C3H/HeJ osteoblasts in part contributes to the differential anabolic response to fluid shear. J Biol Chem.

[33] Tang LL, Wang YL, Pan J, Cai SX (2004). The effect of step-wise increased stretching on rat calvarial osteoblastcollagen production. J Biomech.

[34] Bottlang M, Simnacher M, Schmidt H, Brand RA, Claes L (1997). A cell strain system for small homogeneous strain applications. Biomed Tech.

[35] Owan I, Burr DB, Turner CH, Qiu J, Tu Y, Onyia JE (1997). Mechanotransduction in bone: osteoblasts aremore responsive to fluid forces than mechanical strain. Am J Physiol.

[36] Shirasuna K, Saka M, Hayashido Y, Yoshioka H, Sugiura T, Matsuya T (1993). Extracellular matrix production anddegradation by adenoid cystic carcinoma cells: participation of plasminogen activator and its inhibitor in matrix degradation. Cancer Res.

[37] Guo Y, Zeng QC, Zhang CQ, Zhang XZ, Li RX, Wu JM (2013). Extracellular matrix of mechanically stretched cardiac fibroblasts improves viability and metabolic activity of ventricular cells. Int J Med Sci.

