# Supplementary material for: Integrin-β1, not integrin-β5, mediates osteoblastic differentiation and ECM formation promoted by mechanical tensile strain
Source: Biol Res. 2015 May 14;48(1):25. doi: 10.1186/s40659-015-0014-y (PMC4436743; doi:10.1186/s40659-015-0014-y)
Supplement: Additional file 1: Figure S1. — Preparation of osteoblast ECM which was coated on dishes. A. Osteoblasts were observed via inverted microscopy. The cells were removed after treatment with PBS containing 0.5% Triton X-100 and NH4OH, and treated with 100 units/ml DNase, then the ECMs formed on the surfaces were revealed. B. The cells were stained with DAPI Staining Solution (Wuhan Boster Bioengineering Co., Ltd, Wuhan, China), according to manufacturer’s protocol. Before decellularization, the nucleus were observed with fluorescence microscope. After decellularization, the nucleus were removed. Figure S2. The protein expression of OPN and Runx 2, the ALP activity and mRNA expression of OCN in unstrained cells, and the relative hydroxyproline (Hyp) level of unstrained cells. Pretreatment of integrin-β1 siRNA or integrin-β5 siRNA nearly had no effect on protein expressions of OPN and Runx 2, and the hydroxyproline level. Integrin-β1 siRNA reduced OCN mRNA expression and ALP activity, but the effect was weak. [file 40659_2015_14_MOESM1_ESM.doc]

**Additional file**

## Preparation of osteoblast-ECM

Osteoblasts were derived from MC3T3-E1 cells (clone 4, ATCC catalogue number CRL-2593; ATCC Teddington, UK), a mouse monoclonal pre-osteoblastic cell line that has been shown to differentiate into osteoblasts and osteocytes [1, 2].

The cells were removed according to previous method [3], with some modifications. After washing with PBS, the cells were removed by incubation for 3 min with PBS supplemented with 0.5% Triton X-100 and 10 mM NH_4_OH, then washed three times with PBS. The ECMs attached to the dishes were treated with 100 units/ml DNase (Sigma-Aldrich, St. Louis, MO, USA) for 1 h and the resulting ECM was rinsed with PBS, observed by inverted microscopy ( Figure S1), then stored at 4°C for further use.

**References**

1. Sudo H, Kodama HA, Amagai Y, Yamamoto S, Kasai S: **In vitro differentiation and calcification in a new clonalosteogenic cell line derived from newborn mouse calvaria.** *J Cell Biol* 1983, 96:191–198.

2. Franceschi RT, Iyer BS: **Relationship between collagen synthesis and expression of the osteoblast phenotype in MC3T3-E1 cells**. *J Bone Miner Res* 1992, 7:235–246.

3. Shirasuna K, Saka M, Hayashido Y, Yoshioka H, Sugiura T, Matsuya T: **Extracellular matrix production and degradation by adenoid cystic carcinoma cells: participation of plasminogen activator and its inhibitor in matrix degradation.** *Cancer Res* 1993, 53:147–152.

**
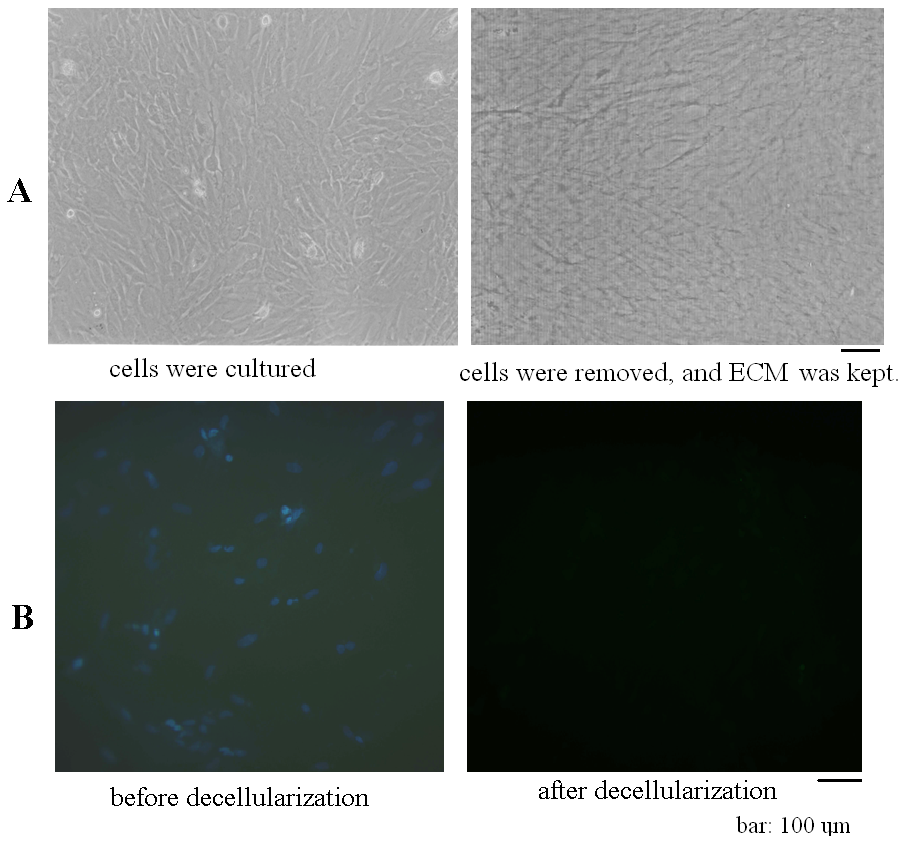
**

**Figure S1 Preparation of osteoblast ECM which was coated on dishes.**

A. Osteoblasts were observed via inverted microscopy. The cells were removed after treatment with PBS containing 0.5% Triton X-100 and 0.10 M NH4OH, and treated with 100 units/ml DNase, then the ECMs formed on the surfaces were revealed.

B. The cells were strained with DAPI Staining Solution (Wuhan Boster Bioengineering Co., Ltd, Wuhan, China), according to manufacturer’s protocol. Before decellularization, the nucleus were observed with fluorescence microscope. After decellularization, the nucleus were removed.

**
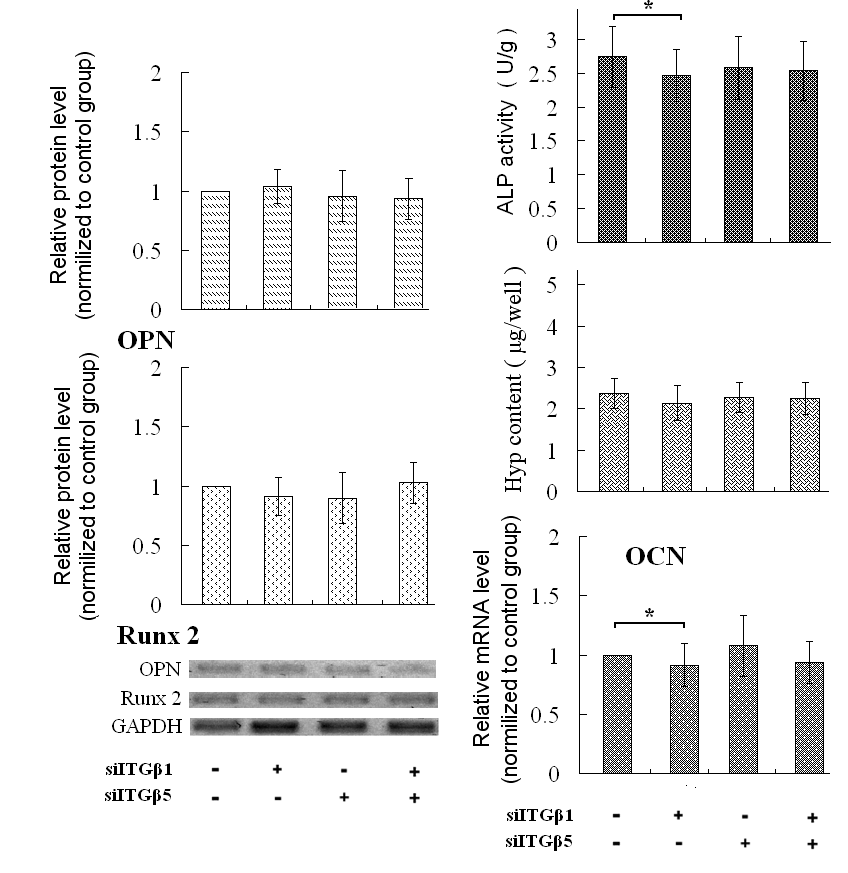
**

**Figure S2 The protein expression of OPN and Runx 2, the ALP activity and mRNA expression of OCN in unstrained cells, and the relative hydroxyproline (Hyp) level of unstrained cells.** Pretreatment of integrin-β1 siRNA or integrin-β5 siRNA nearly had no effect on protein expressions of OPN and Runx 2, and the hydroxyproline level. Integrin-β1 siRNA reduced OCN mRNA expression and ALP activity, but the effect was weak.
